# Crystal structure of 2-methyl-*N*-[(4-methyl­pyridin-2-yl)carbamo­thio­yl]benzamide

**DOI:** 10.1107/S2056989015007860

**Published:** 2015-04-30

**Authors:** Nadiah Ameram, Farook Adam, Nur Nadia Fatihah, Salih Al-Juaid

**Affiliations:** a School of Chemical Sciences, Universiti Sains Malaysia, 11800 Georgetown, Penang, Malaysia; b Chemistry Department, Faculty of Science, King Abdulaziz University, Jeddah, Kingdom of Saudi Arabia

**Keywords:** crystal structure, carbonyl thio­urea, benzamide group, *ortho* position

## Abstract

In the title compound, C_15_H_15_N_3_OS, there is an intra­molecular N—H⋯O hydrogen bond and an intra­molecular C—H⋯S hydrogen bond involving the C=O and C=S bonds which lie on opposite sides of the mol­ecule. The mol­ecule is non-planar with the benzene and pyridine rings being inclined to one another by 26.86 (9)°. In the crystal, mol­ecules are linked by pairs of N—H⋯S hydrogen bonds, forming inversion dimers with an *R*_2_^2^(8) ring motif. The dimers are linked *via* C—H⋯S hydrogen bonds, forming slabs parallel to the *bc* plane.

## Related literature   

For the crystal structures of related compounds, see: Adam *et al.* (2014 ▸, 2015 ▸).
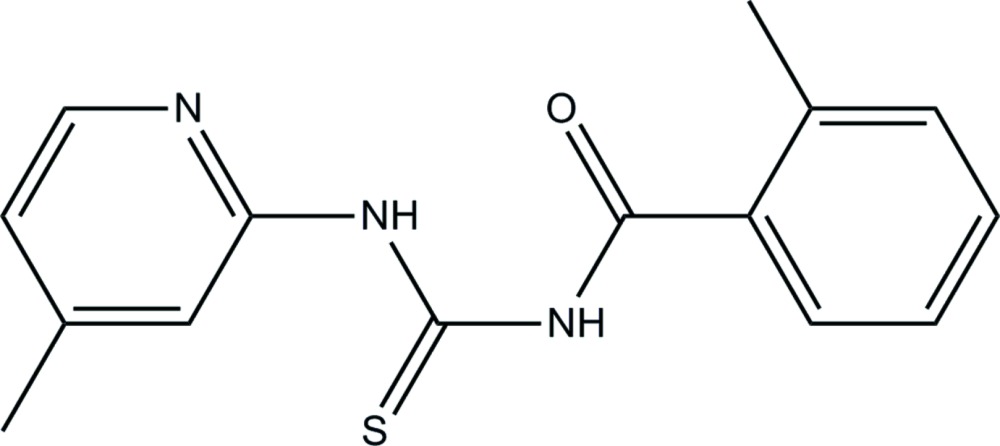


## Experimental   

### Crystal data  

C_15_H_15_N_3_OS
*M* *_r_* = 285.36
Monoclinic, 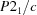

*a* = 11.7131 (3) Å
*b* = 6.2423 (2) Å
*c* = 19.5376 (5) Å
β = 95.312 (2)°
*V* = 1422.39 (7) Å^3^
*Z* = 4
Mo *K*α radiation
μ = 0.23 mm^−1^
*T* = 100 K
0.54 × 0.28 × 0.18 mm

### Data collection  

Bruker SMART APEXII CCD area-detector diffractometer
Absorption correction: multi-scan (*SADABS*; Bruker, 2009 ▸) *T*_min_ = 0.865, *T*_max_ = 0.960
15037 measured reflections
3780 independent reflections
3112 reflections with *I* > 2σ(*I*)
*R*_int_ = 0.031

### Refinement  

*R*[*F* ^2^ > 2σ(*F* ^2^)] = 0.037
*wR*(*F* ^2^) = 0.093
*S* = 1.05
3780 reflections
191 parameters
H atoms treated by a mixture of independent and constrained refinement
Δρ_max_ = 0.32 e Å^−3^
Δρ_min_ = −0.23 e Å^−3^

### 

Data collection: *APEX2* (Bruker, 2009 ▸); cell refinement: *SAINT* (Bruker, 2009 ▸); data reduction: *SAINT*; program(s) used to solve structure: *SHELXS2013* (Sheldrick, 2008 ▸); program(s) used to refine structure: *SHELXL2014* (Sheldrick, 2015 ▸); molecular graphics: *SHELXTL* (Sheldrick, 2008 ▸); software used to prepare material for publication: *SHELXL2014* and *PLATON* (Spek, 2009 ▸).

## Supplementary Material

Crystal structure: contains datablock(s) I. DOI: 10.1107/S2056989015007860/su5119sup1.cif

Structure factors: contains datablock(s) I. DOI: 10.1107/S2056989015007860/su5119Isup2.hkl

Supporting information file. DOI: 10.1107/S2056989015007860/su5119Isup3.cml

**Fig. 1.**
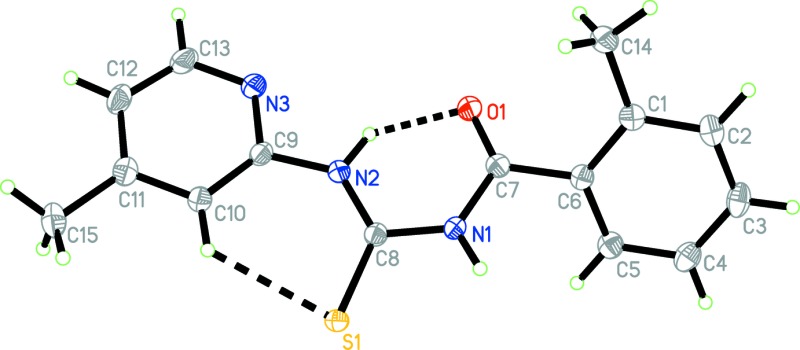
A view of the molecular structure of the title compound, showing the atom labellling. Displacement ellipsoids are drawn at the 50% probability level.

**Fig. 2.**
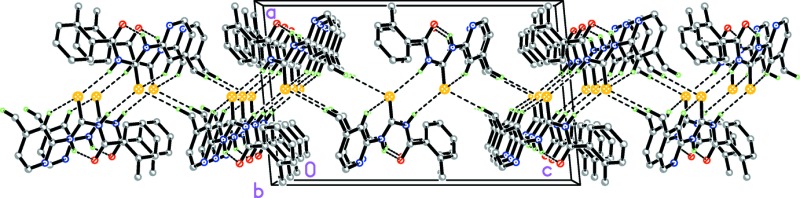
A view along the b axis of the crystal packing of the title compound. Hydrogen bonds are shown as dashed lines (see Table 1 for details).

. DOI: 10.1107/S2056989015007860/su5119fig1.tif
A view of the mol­ecular structure of the title compound, showing the atom labellling. Displacement ellipsoids are drawn at the 50% probability level.

b . DOI: 10.1107/S2056989015007860/su5119fig2.tif
A view along the *b* axis of the crystal packing of the title compound. Hydrogen bonds are shown as dashed lines (see Table 1 for details).

CCDC reference: 974439

Additional supporting information:  crystallographic information; 3D view; checkCIF report

## Figures and Tables

**Table 1:** Hydrogen-bond geometry (, )

*D*H*A*	*D*H	H*A*	*D* *A*	*D*H*A*
N2H1*N*2O1	0.905(17)	1.863(18)	2.6370(16)	142.2(15)
C10H10*A*S1	0.95	2.54	3.2084(14)	127
N1H1*N*1S1^i^	0.891(18)	2.536(18)	3.4046(11)	165.1(16)
C15H15*B*S1^ii^	0.98	2.85	3.8248(16)	175

Symmetry codes: (i) 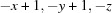; (ii) 
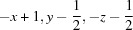
.

## supplementary crystallographic information

### S1. Structural commentary

The title compound shows the bond lengths and angles which are generally normal in *N*-alkyl-*N*'-benzoyl­thio­urea compounds. The bond length of the carbonyl [C8—O1 = 1.229 (15) Å] group of the compound have typical double-bond character, as shown in two closely related compounds, *viz*. 4-methyl-*N*-[(4-methyl­pyridin-2-yl)carbamo­thioyl]benzamide (Adam *et al.*, 2015) and 4-methyl-*N*-[2-(pyridin-2-yl)ethyl­carbamo­thioyl]benzamide (Adam *et al.*, 2014). However, the thio­carbonyl­group [C9—S1 = 1.6755 (13) Å] is longer than the typical C═S of 1.660 (2) Å. The C—N bond lengths are all shorter than the average single C—N bond length of 1.472 (5) Å, being C8—N1 = 1.3930 (16) Å, C9—N1 = 1.3915 (16) Å, C10—N3 = 1.3411 (16) Å and C10—N2 = 1.4157 (16) Å, thus showing varying degrees of single-bond character. There are two intra­molecular hydrogen bonds, *viz.* C11—H11–S1 and N2—H2–O1, which have lengths of 3.2051 (13) and 2.6372 (14) Å, respectively (Table 1). The bond character of the structure is presumed to be the result of the intra­molecular hydrogen bonding that `locks' the molecule into a pseudo-planar six-membered ring structure, similar to structure of the 4-methyl derivative mentioned above (Adam *et al.*, 2015). These results are in agreement with the expected delocalisation in the compound and confirmed by bond angles C9—N2—C10 = 132.02 (11)° and C9—N1—C8 = 128.54 (10)°, indicating *sp*^2^ hybridization of atoms N1 and N2.

In the crystal, molecules are linked by a pair of N—H···S hydrogen bonds forming inversion dimers with an *R*_2_^2^(8) ring motif (Table 1 and Fig. 2). This situation is similar to that in the 4-methyl derivative mentioned above (Adam *et al.*, 2015). The dimers are linked *via* C—H···S hydrogen bonds forming slabs parallel to the *bc* plane.

### S2. Synthesis and crystallization

*o*-Benzoyl chloride (13 mmol) was added dropwise to a stirred acetone solution (30 ml) of ammonium thio­cyanate (13 mmol). The mixture was stirred for 10 min. A solution of 2-amino-4-picoline in acetone was added and the reaction mixture was refluxed for 3 h, after which the solution was poured into a beaker containing some ice cubes. The resulting precipitate was collected by titration, washed several times with a cold ethanol/water mixture and purified by recrystallization from an ethanol solution (m.p. 437.9–438.8 K). FT–IR (KBr, cm^-1^) analysis shows the following vibrational frequencies for N—H, C═O, C—N and C═S at 3237, 1683, 1329 and 1157, respectively. ^1^H NMR results show chemical shifts at 9.545 and 13.501 p.p.m. for the two N—H protons.

### S3. Refinement

Crystal data, data collection and structure refinement details are summarized in Table 2. The N—H H atoms were located in a difference Fourier map and freely refined. The C-bound H atoms were positioned geometrically and refined using a riding model, with C—H = 0.93–0.96 Å and with *U*_iso_(H) = 1.5*U*_eq_(C) for methyl H atoms and 1.2*U*_eq_(C) for other H atoms.

### Crystal data

**Table:**

C_15_H_15_N_3_OS	*F*(000) = 600
*M**_r_* = 285.36	*D*_x_ = 1.333 Mg m^−^^3^
Monoclinic, *P*2_1_/*c*	Mo *K*α radiation, λ = 0.71073 Å
*a* = 11.7131 (3) Å	Cell parameters from 5643 reflections
*b* = 6.2423 (2) Å	θ = 2.9–30.0°
*c* = 19.5376 (5) Å	µ = 0.23 mm^−^^1^
β = 95.312 (2)°	*T* = 100 K
*V* = 1422.39 (7) Å^3^	Block, colourless
*Z* = 4	0.54 × 0.28 × 0.18 mm

### Data collection

**Table:**

Bruker SMART APEXII CCD area-detector diffractometer	3780 independent reflections
Radiation source: fine-focus sealed tube	3112 reflections with *I* > 2σ(*I*)
Graphite monochromator	*R*_int_ = 0.031
φ and ω scans	θ_max_ = 29.0°, θ_min_ = 1.8°
Absorption correction: multi-scan (*SADABS*; Bruker, 2009)	*h* = −15→15
*T*_min_ = 0.865, *T*_max_ = 0.960	*k* = −8→8
15037 measured reflections	*l* = −26→26

### Refinement

**Table:**

Refinement on *F*^2^	0 restraints
Least-squares matrix: full	Hydrogen site location: mixed
*R*[*F*^2^ > 2σ(*F*^2^)] = 0.037	H atoms treated by a mixture of independent and constrained refinement
*wR*(*F*^2^) = 0.093	*w* = 1/[σ^2^(*F*_o_^2^) + (0.0369*P*)^2^ + 0.6623*P*] where *P* = (*F*_o_^2^ + 2*F*_c_^2^)/3
*S* = 1.05	(Δ/σ)_max_ < 0.001
3780 reflections	Δρ_max_ = 0.32 e Å^−^^3^
191 parameters	Δρ_min_ = −0.23 e Å^−^^3^

### Special details

**Table:**

Geometry. All e.s.d.'s (except the e.s.d. in the dihedral angle between two l.s. planes) are estimated using the full covariance matrix. The cell e.s.d.'s are taken into account individually in the estimation of e.s.d.'s in distances, angles and torsion angles; correlations between e.s.d.'s in cell parameters are only used when they are defined by crystal symmetry. An approximate (isotropic) treatment of cell e.s.d.'s is used for estimating e.s.d.'s involving l.s. planes.

### Fractional atomic coordinates and isotropic or equivalent isotropic displacement parameters (Å^2^)

**Table:**

	*x*	*y*	*z*	*U*_iso_*/*U*_eq_	
S1	0.47895 (3)	0.33500 (6)	−0.09032 (2)	0.01997 (10)	
O1	0.13144 (8)	0.65482 (17)	−0.07451 (5)	0.0234 (2)	
N1	0.32328 (9)	0.59313 (19)	−0.04676 (5)	0.0176 (2)	
N2	0.25172 (9)	0.3533 (2)	−0.12933 (6)	0.0193 (2)	
N3	0.12736 (10)	0.1758 (2)	−0.20438 (6)	0.0239 (3)	
C1	0.13792 (11)	0.8962 (2)	0.05748 (6)	0.0195 (3)	
C2	0.14708 (12)	1.0676 (2)	0.10348 (7)	0.0233 (3)	
H2A	0.0875	1.0906	0.1325	0.028*	
C3	0.24044 (13)	1.2056 (2)	0.10816 (7)	0.0260 (3)	
H3A	0.2440	1.3210	0.1400	0.031*	
C4	0.32857 (13)	1.1749 (2)	0.06622 (7)	0.0248 (3)	
H4A	0.3930	1.2679	0.0695	0.030*	
C5	0.32134 (11)	1.0070 (2)	0.01962 (7)	0.0216 (3)	
H5A	0.3811	0.9859	−0.0094	0.026*	
C6	0.22749 (11)	0.8685 (2)	0.01472 (6)	0.0179 (3)	
C7	0.22010 (11)	0.6972 (2)	−0.03910 (6)	0.0179 (3)	
C8	0.34405 (10)	0.4251 (2)	−0.09059 (6)	0.0171 (3)	
C9	0.23574 (11)	0.1778 (2)	−0.17521 (6)	0.0190 (3)	
C10	0.31741 (11)	0.0251 (2)	−0.18797 (7)	0.0205 (3)	
H10A	0.3934	0.0348	−0.1665	0.025*	
C11	0.28585 (12)	−0.1427 (2)	−0.23287 (7)	0.0218 (3)	
C12	0.17278 (13)	−0.1482 (3)	−0.26237 (7)	0.0266 (3)	
H12A	0.1474	−0.2611	−0.2926	0.032*	
C13	0.09824 (12)	0.0126 (3)	−0.24707 (7)	0.0290 (3)	
H13A	0.0218	0.0073	−0.2680	0.035*	
C14	0.03709 (12)	0.7467 (3)	0.05661 (7)	0.0247 (3)	
H14A	−0.0059	0.7766	0.0962	0.037*	
H14B	0.0644	0.5983	0.0589	0.037*	
H14C	−0.0129	0.7678	0.0141	0.037*	
C15	0.37213 (13)	−0.3085 (3)	−0.24966 (8)	0.0282 (3)	
H15A	0.4294	−0.3266	−0.2103	0.042*	
H15B	0.4101	−0.2618	−0.2897	0.042*	
H15C	0.3331	−0.4452	−0.2599	0.042*	
H1N1	0.3827 (15)	0.625 (3)	−0.0167 (9)	0.035 (5)*	
H1N2	0.1874 (14)	0.430 (3)	−0.1245 (8)	0.029 (4)*	

### Atomic displacement parameters (Å^2^)

**Table:**

	*U*^11^	*U*^22^	*U*^33^	*U*^12^	*U*^13^	*U*^23^
S1	0.01482 (15)	0.0247 (2)	0.02041 (15)	−0.00071 (13)	0.00180 (11)	−0.00555 (13)
O1	0.0187 (4)	0.0267 (6)	0.0242 (5)	0.0014 (4)	−0.0009 (4)	−0.0061 (4)
N1	0.0162 (5)	0.0173 (6)	0.0190 (5)	−0.0010 (4)	0.0000 (4)	−0.0034 (4)
N2	0.0157 (5)	0.0211 (6)	0.0209 (5)	0.0004 (5)	0.0007 (4)	−0.0050 (5)
N3	0.0199 (5)	0.0290 (7)	0.0225 (5)	−0.0020 (5)	0.0001 (4)	−0.0070 (5)
C1	0.0210 (6)	0.0193 (7)	0.0178 (6)	0.0018 (5)	−0.0001 (5)	0.0028 (5)
C2	0.0296 (7)	0.0214 (7)	0.0190 (6)	0.0051 (6)	0.0034 (5)	0.0002 (6)
C3	0.0394 (8)	0.0160 (7)	0.0220 (6)	0.0017 (6)	−0.0008 (6)	−0.0020 (5)
C4	0.0304 (7)	0.0160 (7)	0.0275 (7)	−0.0041 (6)	0.0002 (5)	0.0007 (6)
C5	0.0246 (6)	0.0163 (7)	0.0243 (6)	−0.0007 (5)	0.0034 (5)	0.0012 (5)
C6	0.0204 (6)	0.0148 (6)	0.0184 (5)	0.0014 (5)	0.0004 (4)	0.0015 (5)
C7	0.0189 (6)	0.0161 (7)	0.0189 (5)	−0.0004 (5)	0.0035 (4)	0.0016 (5)
C8	0.0179 (6)	0.0173 (7)	0.0165 (5)	−0.0017 (5)	0.0026 (4)	−0.0001 (5)
C9	0.0199 (6)	0.0200 (7)	0.0173 (5)	−0.0045 (5)	0.0026 (4)	−0.0027 (5)
C10	0.0205 (6)	0.0203 (7)	0.0209 (6)	−0.0010 (5)	0.0027 (5)	−0.0024 (5)
C11	0.0292 (7)	0.0200 (7)	0.0172 (6)	−0.0037 (6)	0.0072 (5)	0.0002 (5)
C12	0.0325 (7)	0.0265 (8)	0.0211 (6)	−0.0078 (6)	0.0036 (5)	−0.0077 (6)
C13	0.0242 (7)	0.0363 (9)	0.0259 (7)	−0.0075 (7)	−0.0008 (5)	−0.0098 (7)
C14	0.0231 (6)	0.0287 (8)	0.0229 (6)	−0.0014 (6)	0.0051 (5)	−0.0014 (6)
C15	0.0384 (8)	0.0214 (8)	0.0261 (7)	0.0016 (6)	0.0090 (6)	−0.0033 (6)

### Geometric parameters (Å, º)

**Table:**

S1—C8	1.6768 (13)	C4—H4A	0.9500
O1—C7	1.2223 (15)	C5—C6	1.3949 (19)
N1—C8	1.3898 (17)	C5—H5A	0.9500
N1—C7	1.3924 (16)	C6—C7	1.4964 (18)
N1—H1N1	0.891 (18)	C9—C10	1.3895 (19)
N2—C8	1.3386 (16)	C10—C11	1.3940 (19)
N2—C9	1.4166 (17)	C10—H10A	0.9500
N2—H1N2	0.903 (17)	C11—C12	1.3949 (19)
N3—C13	1.3406 (19)	C11—C15	1.504 (2)
N3—C9	1.3426 (16)	C12—C13	1.381 (2)
C1—C2	1.395 (2)	C12—H12A	0.9500
C1—C6	1.4110 (18)	C13—H13A	0.9500
C1—C14	1.5041 (19)	C14—H14A	0.9800
C2—C3	1.389 (2)	C14—H14B	0.9800
C2—H2A	0.9500	C14—H14C	0.9800
C3—C4	1.389 (2)	C15—H15A	0.9800
C3—H3A	0.9500	C15—H15B	0.9800
C4—C5	1.386 (2)	C15—H15C	0.9800
			
C8—N1—C7	128.43 (11)	N2—C8—S1	126.96 (11)
C8—N1—H1N1	113.8 (12)	N1—C8—S1	118.02 (9)
C7—N1—H1N1	117.3 (12)	N3—C9—C10	123.84 (12)
C8—N2—C9	132.00 (12)	N3—C9—N2	109.89 (12)
C8—N2—H1N2	113.7 (11)	C10—C9—N2	126.25 (12)
C9—N2—H1N2	114.3 (11)	C9—C10—C11	118.80 (12)
C13—N3—C9	116.57 (13)	C9—C10—H10A	120.6
C2—C1—C6	117.29 (13)	C11—C10—H10A	120.6
C2—C1—C14	119.77 (12)	C10—C11—C12	117.68 (13)
C6—C1—C14	122.91 (12)	C10—C11—C15	120.66 (13)
C3—C2—C1	122.11 (13)	C12—C11—C15	121.65 (13)
C3—C2—H2A	118.9	C13—C12—C11	119.21 (13)
C1—C2—H2A	118.9	C13—C12—H12A	120.4
C2—C3—C4	119.94 (13)	C11—C12—H12A	120.4
C2—C3—H3A	120.0	N3—C13—C12	123.87 (13)
C4—C3—H3A	120.0	N3—C13—H13A	118.1
C5—C4—C3	119.22 (13)	C12—C13—H13A	118.1
C5—C4—H4A	120.4	C1—C14—H14A	109.5
C3—C4—H4A	120.4	C1—C14—H14B	109.5
C4—C5—C6	120.92 (13)	H14A—C14—H14B	109.5
C4—C5—H5A	119.5	C1—C14—H14C	109.5
C6—C5—H5A	119.5	H14A—C14—H14C	109.5
C5—C6—C1	120.51 (12)	H14B—C14—H14C	109.5
C5—C6—C7	119.01 (12)	C11—C15—H15A	109.5
C1—C6—C7	120.39 (12)	C11—C15—H15B	109.5
O1—C7—N1	122.59 (12)	H15A—C15—H15B	109.5
O1—C7—C6	122.90 (12)	C11—C15—H15C	109.5
N1—C7—C6	114.49 (11)	H15A—C15—H15C	109.5
N2—C8—N1	115.02 (11)	H15B—C15—H15C	109.5
			
C6—C1—C2—C3	−0.6 (2)	C9—N2—C8—N1	−174.26 (13)
C14—C1—C2—C3	177.60 (13)	C9—N2—C8—S1	6.2 (2)
C1—C2—C3—C4	−0.1 (2)	C7—N1—C8—N2	−1.2 (2)
C2—C3—C4—C5	0.6 (2)	C7—N1—C8—S1	178.38 (11)
C3—C4—C5—C6	−0.4 (2)	C13—N3—C9—C10	1.3 (2)
C4—C5—C6—C1	−0.3 (2)	C13—N3—C9—N2	−177.29 (12)
C4—C5—C6—C7	176.50 (12)	C8—N2—C9—N3	−177.61 (14)
C2—C1—C6—C5	0.77 (19)	C8—N2—C9—C10	3.9 (2)
C14—C1—C6—C5	−177.37 (12)	N3—C9—C10—C11	−0.9 (2)
C2—C1—C6—C7	−175.96 (12)	N2—C9—C10—C11	177.46 (13)
C14—C1—C6—C7	5.90 (19)	C9—C10—C11—C12	−0.4 (2)
C8—N1—C7—O1	−3.2 (2)	C9—C10—C11—C15	178.39 (13)
C8—N1—C7—C6	178.21 (12)	C10—C11—C12—C13	1.1 (2)
C5—C6—C7—O1	−136.49 (14)	C15—C11—C12—C13	−177.63 (14)
C1—C6—C7—O1	40.29 (19)	C9—N3—C13—C12	−0.5 (2)
C5—C6—C7—N1	42.05 (17)	C11—C12—C13—N3	−0.7 (2)
C1—C6—C7—N1	−141.17 (12)		

### Hydrogen-bond geometry (Å, º)

**Table:**

*D*—H···*A*	*D*—H	H···*A*	*D*···*A*	*D*—H···*A*
N2—H1*N*2···O1	0.905 (17)	1.863 (18)	2.6370 (16)	142.2 (15)
C10—H10*A*···S1	0.95	2.54	3.2084 (14)	127
N1—H1*N*1···S1^i^	0.891 (18)	2.536 (18)	3.4046 (11)	165.1 (16)
C15—H15*B*···S1^ii^	0.98	2.85	3.8248 (16)	175

Symmetry codes: (i) −*x*+1, −*y*+1, −*z*; (ii) −*x*+1, *y*−1/2, −*z*−1/2.
