# Supplementary figures and images for: Crystal structure of 2-methyl-N-[(4-methyl­pyridin-2-yl)carbamo­thio­yl]benzamide
Source: Acta Crystallogr E Crystallogr Commun. 2015 Apr 30;71(Pt 5):o356. doi: 10.1107/S2056989015007860 (PMC4420090; doi:10.1107/S2056989015007860)

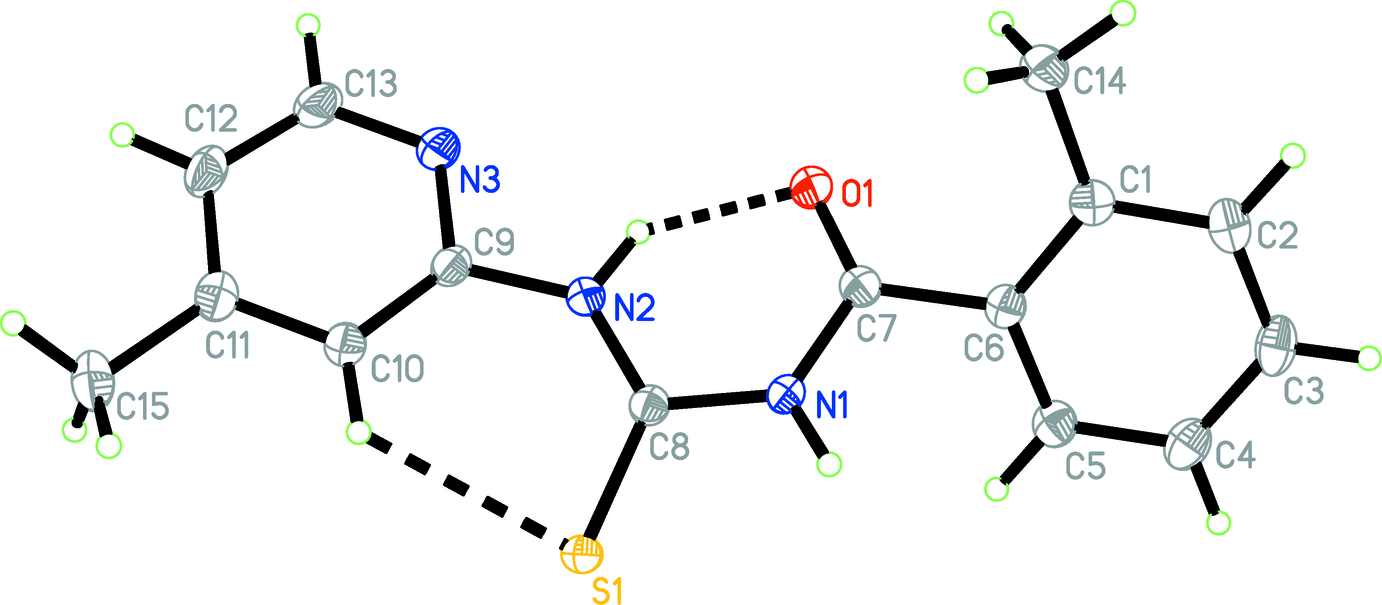

Supplement: Supplementary file 4 [file e-71-0o356-fig1.tif]

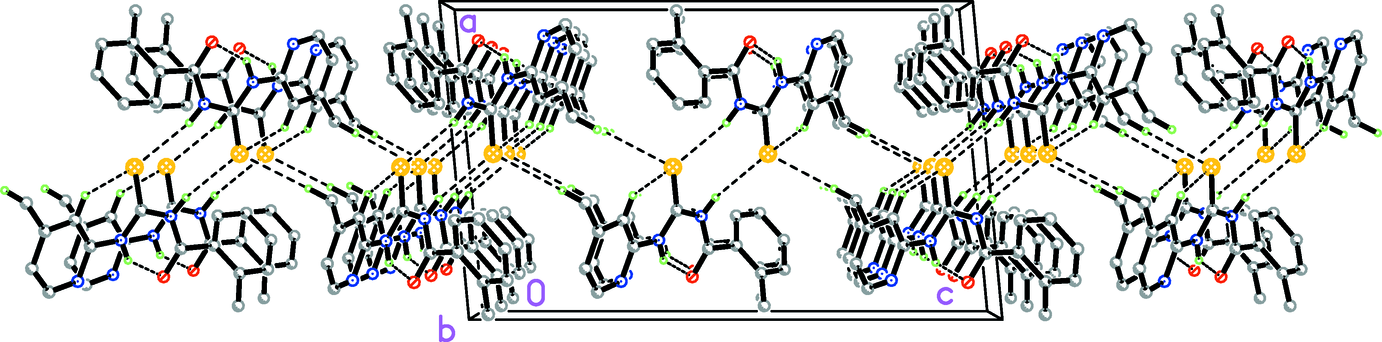

Supplement: Supplementary file 5 [file e-71-0o356-fig2.tif]
